# Optimization of the fermentation process and antioxidant activity of mixed lactic acid bacteria for honeysuckle beverage

**DOI:** 10.3389/fmicb.2024.1364448

**Published:** 2024-04-02

**Authors:** Junjian Ran, Yuhan Tang, Weize Mao, Xia Meng, Lingxia Jiao, Yongchao Li, Ruixiang Zhao, Haoyu Zhou

**Affiliations:** ^1^School of Food Science, School of Life Sciences, Henan Institute of Science and Technology, Henan International Joint Laboratory of Plant Genetic Improvement and Soil Remediation, Xinxiang Engineering Technology Research Center for Agricultural Products Processing, Research and Experimental Base for Traditional Specialty Meat Processing Techniques of the Ministry of Agriculture and Rural Affairs of the People's Republic of China, Xinxiang, China; ^2^School of Food Engineering, Xinxiang Institute of Engineering, Xinxiang, China; ^3^College of Pharmacy, Xinxiang University, Xinxiang, China

**Keywords:** honeysuckle, lactic acid bacteria fermentation, orthogonal test, antioxidant activity, sensory evaluation

## Abstract

The aim of the research was to obtain a high healthcare honeysuckle beverage with strong antioxidant activity. Honeysuckle (*Lonicera japonica Thunb*) was used as the raw material in this experiment. The effects of fermentation temperature, fermentation time, lactic acid bacteria inoculation amount, and sugar addition amount on the sensory quality of honeysuckle beverage were investigated by single factor test and orthogonal test, and the best process was obtained. The physicochemical indexes and antioxidant activity of honeysuckle beverages fermented with lactic acid bacteria were studied. The results showed that the fermentation temperature of the beverage was 37 °C, the fermentation time was 24 h, the inoculation amount of *Lactiplantibacillus plantarum* and *Lactobacillus acidophilus* mixed starter (1:1) was 3%, and 8% white granulated sugar was added. The highest sensory score was 87.30 ± 0.17, which was the optimal process. The honeysuckle liquid mixed inoculation with *Lactiplantibacillus plantarum* and *Lactobacillus acidophilus* was fermented for 24 h. The number of viable bacteria reached 9.84 ± 0.02 lg cfu/mL, the pH value was 3.10 ± 0.01, and the total polyphenol content was 7.53 ± 0.03 mg GAE/g. The number of lactic acid bacteria, pH, total polyphenol content, and free radical scavenging rate were significantly increased (*p* < 0.05) compared with the non-inoculated and single-inoculated lactic acid bacteria. To sum up, it was concluded that a better quality beverage could be obtained by fermenting a solution of honeysuckle with *Lactiplantibacillus plantarum* and *Lactobacillus acidophilus* mixed fermentation agent, providing a new approach and new ideas for the development of deep processing and fermented beverages using honeysuckle.

## 1 Introduction

In recent years, functional foods have attracted more and more attention due to their health-promoting benefits. Common functional foods include probiotic foods and beverages (Gao et al., 2019), and functional drinks are defined as non-alcoholic beverages with herbs, vitamins, minerals, amino acids, or probiotics added (Giri et al., 2018). Probiotics are beneficial bacteria that can have therapeutic effects on the host when ingested in sufficient quantities, and probiotics occupy an important position in the whole functional food market. The development of new probiotic beverages mainly depends on whether probiotics can represent a sufficient number of living cells, which is conducive to adapting to the host’s intestinal microbiota (Islam et al., 2021). Many health benefits have the consumption of probiotics, such as maintaining disturbed gut microbiota, reducing lactose intolerance, improving digestion, and strengthening the immune system (Giri et al., 2018; Chen et al., 2020; Dahiya and Nigam, 2023).

Honeysuckle (*Lonicera japonica Thunb*) is the dry bud or initial flower of the honeysuckle family (Fan et al., 2019). It has been widely used in medicine and food treatment since ancient times. Honeysuckle products are widely used in traditional Chinese medicine, and it is considered to be a promising material in a variety of pharmacology, cosmetics, food industry, and agriculture (Orsavová et al., 2022). Studies have found that honeysuckle contains a variety of antioxidant and anti-tumor compounds such as phenolic acids, flavonoids, triterpenoid saponins, polysaccharides, and volatile oils (Cui et al., 2014), which are beneficial to human health. Studies have shown that honeysuckle has anti-cancer, anti-inflammatory, anti-oxidation, and other effects (Cai et al., 2019, 2022; Su et al., 2019).

Lactic acid bacteria (LAB) are gram-positive (Zhang et al., 2018) and have many probiotic functions such as regulating the metabolism of intestinal microorganisms and promoting the reproduction of intestinal beneficial bacteria (Wang et al., 2017). Their probiotic function has an inseparable relationship with people’s health. LAB play multiple roles in agriculture, food, and clinical fields (Ayivi et al., 2020). It has a strong acid production ability and is the main starter for making yogurt and other foods, and it is the most widely used *Lactobacillus* and related genera (Singhal et al., 2021). LAB beverages promote digestion and absorption, especially suitable for the elderly and children, with great potential for biotechnology (Mokoena, 2017). The application of LAB in the fermentation of honeysuckle beverage can prolong the shelf life of honeysuckle beverage, increase the types of honeysuckle beverage, and meet the market demand of different consumers. Excellent scavengers of reactive oxygen species and nitrogen species are phenolic compounds in blue honeysuckle, such as anthocyanin, chlorogenic acid, quercetin, or kaempferol (Bonarska-Kujawa et al., 2014). The results showed that the fermentation of bayberry pomace by different kinds of probiotics could increase the content of total polyphenols, flavonoids, and polysaccharides in bayberry pomace, so as to improve its antioxidant activity (Zhu et al., 2020). Some scholars have proved that LAB can convert the bound phenolic substances in honeysuckle that are not easily digested and absorbed by the human body into free phenolic substances that can be digested and utilized by the human body.

In this study, honeysuckle was used as raw material. The fermentation temperature, fermentation time, inoculation amount of *Lactiplantibacillus plantarum* and *Lactobacillus acidophilus* mixed starter (1:1), and the addition amount of white granulated sugar were investigated by single factor test and orthogonal test to determine the best optimization process. The pH value, number of viable bacteria, and antioxidant activity of honeysuckle liquid during LAB fermentation were studied. The results provided technical parameters and theoretical basis for improving the deep processing of honeysuckle-fermented beverage, so as to ensure its stability and reliability in the production process.

## 2 Materials and methods

### 2.1 Materials and reagents

Honeysuckle and granulated sugar were purchased from local supermarkets; *Lactobacillus acidophilus* zrx02 (GenBank No.MF804413) and *Lactiplantibacillus plantarum* zrx03 (GenBank No.MN784485) were kept by the Food Biotechnology Laboratory of the Food College of Henan Institute of Science and Technology. Gallic acid was purchased from Chengdu Munster Co., Ltd. Folin-phenol and Tris were purchased from Beijing Solarbio Co., Ltd. MRS medium was purchased from Beijing Aoboxing Biotechnology Co., Ltd. The reagents mentioned above are analytically pure.

### 2.2 Process flow of honeysuckle-fermented beverage

The process flow of honeysuckle-fermented beverage is shown in the following diagram (Gao et al., 2019).


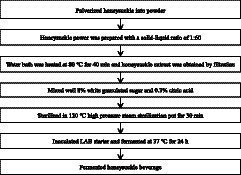


### 2.3 Activation of strain

Two strains of LAB preserved in a −80 °C refrigerator were streaked and cultured. Single colonies were picked in MRS liquid medium and cultured in a shaking table at 37 °C, 180 r/min, and continuous activation for 2–3 times to obtain activated strains. The activated bacterial solution was incubated in MRS liquid medium at 1% inoculum and cultured in a shaking table at 37 °C, 180 r/min until the logarithmic end for reserve.

### 2.4 Single factor experiment

#### 2.4.1 Determination of fermentation temperature

When the honeysuckle extract was cooled to room temperature, the amount of white sugar added was 8%, the amount of citric acid added was 0.3%, the sterilization was 30 min at 121 °C, the amount of *Lactiplantibacillus plantarum* and *Lactobacillus acidophilus* mixed starter (1:1) was 3%, and the fermentation time was 24 h. Under the conditions, the effects of fermentation temperature (31 °C, 34 °C, 37 °C, 40 °C, 43 °C) on the fermented honeysuckle beverage were studied to determine the optimal parameters of fermentation temperature.

#### 2.4.2 Determination of fermentation time

When the honeysuckle extract was cooled to room temperature, the amount of white sugar added was 8%, the amount of citric acid added was 0.3%, the sterilization was 30 min at 121 °C, the inoculation amount of *Lactiplantibacillus plantarum* and *Lactobacillus acidophilus* mixed starter (1:1) was 3%, and the fermentation temperature was 37 °C. Under the conditions, the effect of fermentation time (12 h, 18 h, 24 h, 30 h, 36 h) on the fermented honeysuckle beverage was studied to determine the best time for beverage fermentation.

#### 2.4.3 Determination of inoculation amount

When the honeysuckle extract was cooled to room temperature, the amount of white sugar added was 8%, the amount of citric acid added was 0.3%, the sterilization was 30 min at 121 °C, the fermentation temperature was 37 °C, and the fermentation time was 24 h. Under the conditions, the effects of *Lactiplantibacillus plantarum* and *Lactobacillus acidophilus* mixed starter (1:1) inoculation amounts (1, 2, 3, 4, 5%) on the fermented honeysuckle beverage were studied to determine the optimal inoculation amount of the mixed starter.

#### 2.4.4 Determination of the amount of white sugar added

When the honeysuckle extract was cooled to room temperature, the amount of citric acid added was 0.3%, the sterilization was 30 min at 121 °C, the inoculation amount of *Lactiplantibacillus plantarum* and *Lactobacillus acidophilus* mixed starter (1:1) was 3%, the fermentation temperature was 37 °C, and the fermentation time was 24 h. Under the conditions, the effect of the addition amount of white granulated sugar (2, 4, 8, 12, 16%) on the fermented honeysuckle beverage was studied to determine the optimal amount of white granulated sugar added.

### 2.5 Orthogonal experiment and verification experiment

To comprehensively consider the influence of various factors on the sensory score, based on the experimental results of a single factor, the fermentation temperature A, fermentation time B, the addition amount of mixed starter C, and the addition amount of white sugar D were taken as the investigation factors, and the sensory score of honeysuckle-fermented beverage was taken as the evaluation index. The orthogonal test of four factors and three levels was designed to determine the optimal process parameters, as shown in Table 1.

**Table 1:** Level table of fermentation process factors of honeysuckle flower.

Factor	A (°C)	B (h)	C (mL)	D (g)
Level 1	34	12	2%	4%
Level 2	37	18	3%	8%
Level 3	40	24	4%	12%

### 2.6 Sensory evaluation

The sensory evaluation group consisted of 10 food science and engineering experts, and the sensory evaluation of the fermented honeysuckle beverage was performed. The results were shown as the means ± SD. Sensory evaluation members included five men and five women aged between 25 and 50 years old and often performed sensory evaluation on fermented beverages. All panel members had no food allergies or intolerances and were regular consumers of fermented beverages. Evaluators were encouraged not to drink alcohol or eat spicy food within 12 h before the evaluation, gargling was required during the evaluation, and a 5-min interval was required during the evaluation of different honeysuckle solutions. Sensory evaluation members were informed of the purpose of the study and received written informed consent. All the tested samples were food-grade, and the evaluation system consisted of four parts: color, aroma, shape, and texture. Sensory analysis was carried out by quantitative descriptive analysis: color 0–10 scores (0 = worst, 10 = best); aroma score 0–20 (0 = worst, 20 = best); shape 0–20 scores (0 = worst, 20 = best); texture score 0–50 (0 = worst, 50 = best). The samples were randomly distributed to the members of sensory evaluation, and the analyses were carried out in triplicate. The total score was 100, as shown in Table 2.

**Table 2:** Scoring standard of mixed LAB-fermented honeysuckle beverage.

Factor	Evaluation criteria	Score
Color	The color of the drink is yellow, transparent, and glossy	7–10
	The color of the drink is dark yellow and slightly shiny	4–7
	The color of the drink is dull and dull	< 4
Aroma	The drink has a typical honeysuckle aroma and is relatively refreshing	15–20
	The drink has a light honeysuckle aroma	8–15
	The drink has almost no honeysuckle aroma and a pungent odor	< 8
Shape	The drink is clear and not cloudy	15–20
	The drink is clear and slightly cloudy	8–15
	The drink is cloudy and slightly precipitated	< 8
Texture	The taste is soft, moderate sweet and sour, refreshing	35–50
	The taste is medium, slightly sour or sweet	18–35
	The taste is poor, sour with a sense of stimulation, difficult to accept	< 18

### 2.7 Determination of physical and chemical indexes and antioxidant activity

#### 2.7.1 Determination of viable number of lactic acid bacteria

*Lactiplantibacillus plantarum* and *Lactobacillus acidophilus* are aerotolerant anaerobes. To facilitate the experiment, the aerobic method was used to count LAB plates (Chen et al., 2020). The whole experiment was carried out on a single-sided air supply purification table (SW-CJ-1D, Suzhou Zhi Purification Equipment Co., LTD.). MRS medium (Beijing Aoboxing Biotechnology Co., Ltd) was cultured in a biochemical incubator (SHP-250, Shanghai Sanfa Scientific Instrument Co., LTD.) at 37 °C for 48 h. The honeysuckle liquid was serially diluted, and the dilution multiple of 6, 7, and 8 times was selected as the dilution gradient. The selected dilution gradient sample was coated with 0.1 mL on a plate, and three parallel experiments were performed. The plates were cultured at 37 °C for a certain period of time and then taken out for counting.

#### 2.7.2 Determination of pH value

A pH meter (PHS-3C, Shanghai Sheng Magnetics Co., LTD) was used to determine pH according to the description (Muniandy et al., 2015).

#### 2.7.3 Preparation of polyphenol extract

The honeysuckle liquid obtained by centrifugation (H2050R table top high-speed refrigerated centrifuge, Hunan Xiangyi Laboratory Instrument Development Co., LTD.) was mixed with ethanol (honeysuckle liquid: anhydrous ethanol = 3:7). After alcohol precipitation at 4 °C for 6 h, macromolecular substances such as polysaccharides were removed by centrifugation at 7,100 × g for 1 min. Ultrasonic extraction was performed twice at an ultrasonic power of 900 W, an ultrasonic temperature of 50 °C, and an ultrasonic time of 2 h. The extract was centrifuged twice, and the impurities were filtered and sterilized by an organic filter membrane. The solution was concentrated to 30 mL at 50 °C by rotary evaporation (Brglez Mojzer et al., 2016; Sridhar et al., 2021). Honeysuckle polyphenol extract can be obtained.

#### 2.7.4 Determination of total polyphenol content

Referring to the method of Koczka et al. (2018), the linear regression equation was obtained by the Folin–Ciocalteu colorimetric method: *y* = 0.0983x + 0.0078, *R*_2_ = 0.9982. According to the above method, the sample solution was used instead of gallic acid solution to determine the absorbance. According to the established standard curve, the total polyphenol content of honeysuckle was calculated and its unit was mg GAE/g.

#### 2.7.5 Determination of DPPH free radical scavenging ability

By referring to the determination method (Aloğlu and Öner, 2011), DPPH was weighed to 39.4 mg and filled with anhydrous ethanol into a 500 mL volumetric flask to obtain a 0.2 mmol/L DPPH solution. The sample was stored in the refrigerator at 4 °C and set aside. A total of 4 mL 0.2 mmol/L DPPH solution and 4 mL of sample were taken, and anhydrous ethanol was used instead of the sample solution as blank control. The same amount of anhydrous ethanol was added in another group of sample solutions without adding DPPH solution. After reaction for 30 min in the dark, the absorbance was measured at 517 nm. Each group was tested three times in parallel, and the average value was used to calculate the DPPH free radical scavenging rate. As follows is the calculation formula of the DPPH free radical scavenging rate:


$$x=\frac{1-A_{1}-A_{2}}{A_{0}}\times 100$$

A_0_ represents the absorbance of the DPPH and anhydrous ethanol at 517 nm.

A_1_ represents the absorbance of the DPPH and sample measured at 517 nm.

A_2_ represents the absorbance of the anhydrous ethanol and sample at 517 nm.

#### 2.7.6 Determination of OH free radical scavenging ability

Referring to the determination method (Erdoğan and Özbakır Işın, 2021), 1.0 mL 9 mmol/L salicylic acid-ethanol, 1.0 mL 9 mmol/L FeSO_4_, and 1.0 mL sample were weighed and 1.0 mL 0.8 mmol/L H_2_O_2_ was added. The absorbance measured at 510 nm was denoted as A1. In addition, distilled water was used to replace the sample as the blank group, and the absorbance measured at 510 nm was recorded as A_0_. Distilled water was used instead of H_2_O_2_ as the reaction blank group, and the absorbance measured at 510 nm was recorded as A_2_. Each group was tested three times in parallel, and the average value was used to calculate the OH free radical scavenging rate. As follows is the calculation formula of the OH free radical scavenging rate:


$$x=\frac{\left(A_{0}-A_{1}+A_{2}\right)}{A_{0}}\times 100$$

A_0_ is the absorbance value of the sample blank group.

A_1_ is the absorbance value of the sample group.

A_2_ is the absorbance value of the reaction blank group.

#### 2.7.7 Determination of the scavenging ability of superoxide anion radical

Referring to the determination method given by Li et al. (2021), 1 mL sample solution was taken, and 3 mL Tris–HCl buffer (50 mmol, pH = 8.2) was added to each well. Then, 12 uL pyrogallol solutions (30 mmol/L) were added. After mixing, the reaction was carried out in a water bath at 25 °C for 4 min, then 0.5 mL HCl (8 mol/L) was added to terminate the reaction, and the absorbance A_x_ was determined at 320 nm. Distilled water was used as a blank control. Each group was tested three times in parallel, and the average value was used to calculate the superoxide anion radical free radical scavenging rate. As follows is the calculation formula of superoxide anion radical free radical scavenging rate:


$$x=\left[1-\frac{\left(A_{X}-A_{X0}\right)}{A_{0}}\right]\times 100$$

A_0_ is the absorbance value of the blank control sample.

A_x_ is the absorbance value of the sample solution.

A_x0_ is the absorbance value of the sample solution without color-developing agent pyrogallol.

### 2.8 Data analysis

All experiments were repeated three times. The results were expressed by the mean ± standard deviation. The data were analyzed by IBM SPSS Statistics 26 software, and a *t*-test was performed. The orthogonal design assistant of II V3.1 Professional edition and Origin 2018 was used for mapping. A *p-value of* < 0.05 indicated that there was a significant difference in the statistics.

## 3 Results

### 3.1 Influence of single factor variables on the quality of honeysuckle-fermented beverage

**Figure 1:**
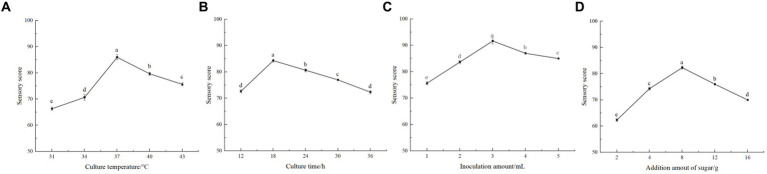
Single factor test. **(A)** is the effect of fermentation temperature on the sensory evaluation of fermented beverages. **(B)** is the effect of fermentation time on the sensory evaluation of fermented beverages. **(C)** is the effect of the inoculation amount of *Lactiplantibacillus plantarum* and *Lactobacillus acidophilus* mixed starter (1:1) on the sensory evaluation of fermented beverages. **(D)** is the effect of the amount of white sugar added on the sensory evaluation of fermented beverages.

As shown in Figure 1A, the sensory scores of honeysuckle-fermented beverage were higher (*p* < 0.05) when the fermentation temperature was between 34 and 40 °C. The beverage had a good aroma and taste and was easily accepted by people. The highest point of 86.00 ± 1.00 was reached when the fermentation temperature gradually increased to 37 °C. The sensory score decreased sharply (*p* < 0.05) when the fermentation temperature continued to rise (*p* < 0.05). It can be seen that low temperature has a certain effect on the activity of LAB, thus inhibiting the fermentation of lactic acid. However, the temperature was too high, leading to the loss of flavor, nutrition, and other substances, thus affecting the quality of fermented beverages. The fermentation temperature was selected as 34 °C, 37 °C, and 40 °C for the orthogonal experiment after comprehensive consideration of various factors.

During the fermentation process, it can be seen from Figure 1B that the sensory rating score was significantly increased (*p* < 0.05) at 12–18 h, reaching a maximum of 84.33 ± 0.58. The sensory score gradually decreased with time and decreased to the lowest at 36 h of fermentation (*p* < 0.05). After 12–24 h fermentation, the product had a higher sensory score, indicating that the beverage had strong fermentation flavor, good taste, and moderate sweetness and sour during this period. During the rest of the fermentation time, the sensory scores of the beverage were not high. It had a certain impact on the taste and reduced the quality of the beverage after the fermentation time exceeded 24 h. Therefore, not only could 12- to 24-h fermentation save fermentation time and costs but it could also obtain the appropriate acidity and flavor. So in the orthogonal test, the three levels of 12 h, 18 h, and 24 h were selected.

The different inoculation amount of mixed starter in fermented beverages also had a great impact on the flavor and taste. As shown in Figure 1C, when the inoculation amount was between 1 and 3%, the sensory evaluation score increased significantly (*p* < 0.05), from 75.67 ± 0.58 to 91.67 ± 1.15. The sensory score of honeysuckle beverage decreased gradually when the inoculation amount was higher than 3%. It can be seen that too low inoculation amount made its fermentation slow. Considering the factors such as product quality and experiment cost, three levels of the addition amount of 2, 3, and 4% were selected for the orthogonal experiment.

As shown in Figure 1D, when the addition of white granulated sugar reached 8%, the beverage sensory score could reach 82.33 ± 0.58, reaching the highest level (*p* < 0.05). When the amount of sugar added was less than 8%, the fermented beverage had a sour taste and a light flavor. The score decreased when the added amount of white granulated sugar exceeded 8%. Perhaps it is because the more sugar added, the higher the osmotic pressure of the fermentation system; thus, it would inhibit the growth of LAB. At the same time, it also made the beverage sweeter and affected the sensory evaluation. It can be seen that the appropriate increase in white granulated sugar to promote the fermentation of LAB makes the taste better. Therefore, the three levels of sugar addition of 4, 8, and 12% were selected for orthogonal experiments.

### 3.2 Orthogonal experiment

According to the range results in Table 3, the sensory score of the beverage was affected by many factors. The primary and secondary order of the influencing factors of the test was A > C > B > D. From the analysis of variance in Table 4, it can be seen that the factors that have a very significant impact on the quality of the beverage were the fermentation temperature and the amount of inoculation, the factors that had a significant impact on the fermentation time, and the amount of white granulated sugar added was not significantly affected. The optimal fermentation combination was A_2_B_3_C_2_D_1_, the fermentation temperature was 37 °C, the fermentation time was 24 h, the inoculation amount of *Lactiplantibacillus plantarum* and *Lactobacillus acidophilus* mixed starter (1:1) was 3%, and the amount of white granulated sugar was 8%.

**Table 3:** Orthogonal experiment results under multiple factors.

Test tube number	A (g/L)	B (h)	C (mL)	D (g)	Sensory score
1	1	1	1	1	77
2	1	2	2	2	79
3	1	3	3	3	80
4	2	1	2	3	83.5
5	2	2	3	1	81
6	2	3	1	2	79.6
7	3	1	3	2	74.9
8	3	2	1	3	70.5
9	3	3	2	1	76.2
k1	78.667	78.467	75.700	78.067	
k2	81.367	76.833	79.567	77.833	
k3	73.867	78.600	78.633	78.000	
*R*-range	7.500	1.767	3.867	0.234	
Primary and Secondary order	A>C>B>D				
Optimal level	A_2_B_3_C_2_D_1_				

**Table 4:** Analysis of variance of orthogonal experiment results.

Factor	Deviation sum of squares	Degree of freedom	*F*-ratio	*F* critical value	Significance
A	86.580	2	995.172	19.000	**
B	5.807	2	66.747	19.000	*
C	24.427	2	280.770	19.000	**
D	0.087	2	1.000	19.000	
Error	0.09	2			

*significant, *p* < 0.05; **extremely significant, *p* < 0.01.

### 3.3 Verification experiment

Since the optimal ratio A_2_B_3_C_2_D_1_ did not appear in the orthogonal test, further verification tests were needed to ensure its reliability. After verification, the sensory score of honeysuckle LAB beverage was 87.30 ± 0.17 under the conditions of fermentation product at 37 °C for 24 h, inoculation amount of 3%, and white sugar addition amount of 8%, which was higher than the optimal result of the orthogonal test. Based on the sensory evaluation of honeysuckle LAB beverage, the optimum production process was obtained as follows: fermentation temperature 37 °C, fermentation time 24 h, *Lactiplantibacillus plantarum* and *Lactobacillus acidophilus* mixed starter (1:1) inoculation amount 3%, white sugar addition amount 8%.

### 3.4 Effects of LAB fermentation on the quality of honeysuckle liquid

#### 3.4.1 Changes in the number of LAB during the fermentation of honeysuckle liquid

**Figure 2:**
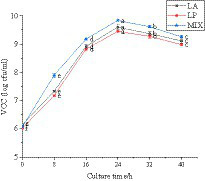
Behavior of LAB during the fermentation process. LA represents honeysuckle liquid inoculated with *Lactobacillus acidophilus*. LP represents honeysuckle liquid inoculated with *Lactiplantibacillus plantarum*. MIX represents honeysuckle liquid inoculated with mixed starter cultures of *Lactiplantibacillus plantarum* and *Lactobacillus acidophilus*. CK represents honeysuckle liquid without LAB inoculation.

The viable count of LAB in the fermentation process of honeysuckle liquid was determined. It can be seen from Figure 2 that the total number of LAB after fermentation of honeysuckle liquid reached more than 6.02 ± 0.03 lg cfu/mL (the total number of colonies in the CK was not shown in the figure). With the longer fermentation time, the total number of LAB colonies increased and reached the highest at 24 h fermentation (*p* < 0.05), all reaching 9.0 lg cfu/mL. The total number of LAB was 9.60 ± 0.08 lg cfu/mL, 9.45 ± 0.04 lg cfu/mL, and 9.84 ± 0.02 lg cfu/mL, respectively. Because when the inoculation amount of LAB is constant, the change in time has a certain influence on the total number of LAB colonies.

#### 3.4.2 Changes in pH during the fermentation of honeysuckle

**Figure 3:**
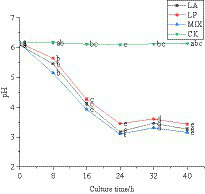
Change in pH value of honeysuckle liquid during fermentation. LA represents honeysuckle liquid inoculated with *Lactobacillus acidophilus*. LP represents honeysuckle liquid inoculated with *Lactiplantibacillus plantarum*. MIX represents honeysuckle liquid inoculated with mixed starter cultures of *Lactiplantibacillus plantarum* and *Lactobacillus acidophilus*. CK represents honeysuckle liquid without LAB inoculation.

The pH value can reflect whether the fermented honeysuckle liquid is normal. In this experiment, the change rule of pH value in the fermentation process of honeysuckle liquid was determined. The pH value of honeysuckle liquid without LAB inoculation remained at approximately 6.12. During 0–24 h of fermentation, the pH value of honeysuckle liquid inoculated with LAB decreased significantly (*p* < 0.05) (Figure 3). At 24–40 h of fermentation, the pH value gradually decreased and tended to be stable (*p* < 0.05). At 24 h of fermentation, the pH value of honeysuckle liquid fermented by LAB reached the lowest value, and the pH value of honeysuckle liquid fermented by mixed starter culture was the smallest, which was 3.10 ± 0.01.

#### 3.4.3 Changes in the total polyphenol content of honeysuckle during the fermentation

**Figure 4:**
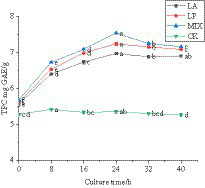
Changes in total polyphenol content during fermentation. LA represents honeysuckle liquid inoculated with *Lactobacillus acidophilus*. LP represents honeysuckle liquid inoculated with *Lactiplantibacillus plantarum*. MIX represents honeysuckle liquid inoculated with mixed starter cultures of *Lactiplantibacillus plantarum* and *Lactobacillus acidophilus*. CK represents honeysuckle liquid without LAB inoculation.

The polyphenol content in the fermentation process was determined. The results are shown in Figure 4, and it can be seen that the total polyphenol content of honeysuckle liquid without LAB remained at approximately 5.32 mg GAE/g during the fermentation process at 37 °C. The total polyphenol content of honeysuckle liquid fermented by LAB was significantly increased (*p* < 0.05), and the honeysuckle liquid fermented by mixed strains was higher than that fermented by single strains. The total polyphenol content reached the highest at 24 h of fermentation, reaching 6.96 ± 0.04 mg GAE/g, 7.23 ± 0.05 mg GAE/g, and 7.53 ± 0.03 mg GAE/g, respectively.

### 3.5 Effects of honeysuckle fermentation on antioxidant activity of honeysuckle beverage

#### 3.5.1 The change in DPPH free radical scavenging ability of honeysuckle during the fermentation

**Figure 5:**
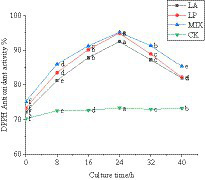
Changes in DPPH free radical clearance in the fermentation process. LA represents honeysuckle liquid inoculated with *Lactobacillus acidophilus*. LP represents honeysuckle liquid inoculated with *Lactiplantibacillus plantarum*. MIX represents honeysuckle liquid inoculated with mixed starter cultures of *Lactiplantibacillus plantarum* and *Lactobacillus acidophilus*. CK represents honeysuckle liquid without LAB inoculation.

Polyphenols are the main active substances that play an antioxidant role. The determination of the antioxidant capacity of unfermented and fermented honeysuckle liquid can reflect the antioxidant capacity of honeysuckle from the side. The single electron of DPPH free radical paired with polyphenol of fermented honeysuckle, which could eliminate DPPH free radical. According to Figure 5, the DPPH free radical scavenging rate of honeysuckle liquid increased significantly (*p* < 0.05). At 24 h of fermentation, the DPPH free radical scavenging ability of honeysuckle liquid fermented by LAB reached the highest value. The fermentation by *Lactobacillus acidophilus* was 92.47 ± 0.03%, the fermentation by *Lactiplantibacillus plantarum* was 94.94 ± 0.02%, and the fermentation by mixed starter was 95.17 ± 0.05%. Compared with before fermentation, it increased by 20.33, 21.65, and 19.99%, respectively (*p* < 0.05).

#### 3.5.2 Changes in OH free radical scavenging ability of honeysuckle during the fermentation

**Figure 6:**
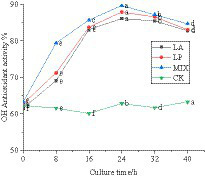
Changes in OH free radical clearance in the fermentation process. LA represents honeysuckle liquid inoculated with *Lactobacillus acidophilus*. LP represents honeysuckle liquid inoculated with *Lactiplantibacillus plantarum*. MIX represents honeysuckle liquid inoculated with mixed starter cultures of *Lactiplantibacillus plantarum* and *Lactobacillus acidophilus*. CK represents honeysuckle liquid without LAB inoculation.

The hydroxyl group reacts with polyphenols in the liquid of honeysuckle to remove the hydroxyl group. The OH free radical scavenging rate of honeysuckle liquid fermented by LAB was significantly improved, which was due to the effect of polyphenols and hydroxyl groups. The scavenging rate of honeysuckle solution without LAB on OH free radical remained at approximately 61.95%. During the fermentation process, the scavenging of OH radicals by honeysuckle liquid fermented by LAB was significantly improved (*p* < 0.05) (Figure 6).

#### 3.5.3 Changes in the superoxide anion radical scavenging ability of honeysuckle during the fermentation

**Figure 7:**
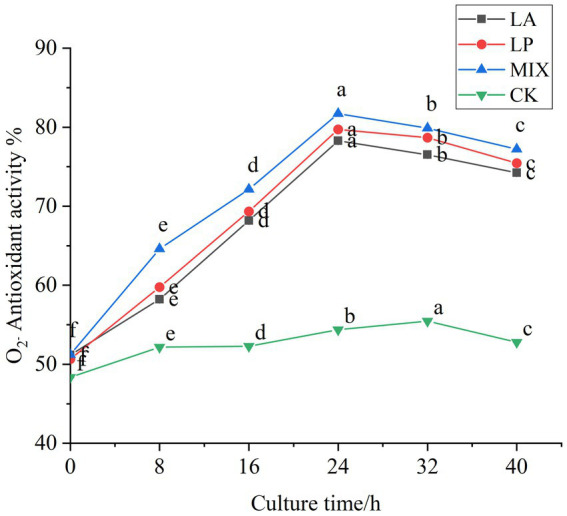
Changes in superoxide anion radical clearance in the fermentation process. LA represents honeysuckle liquid inoculated with *Lactobacillus acidophilus*. LP represents honeysuckle liquid inoculated with *Lactiplantibacillus plantarum*. MIX represents honeysuckle liquid inoculated with mixed starter cultures of *Lactiplantibacillus plantarum* and *Lactobacillus acidophilus*. CK represents honeysuckle liquid without LAB inoculation.

The scavenging ability of superoxide anion free radical during fermentation was determined. In the first 24 h of fermentation, the scavenging rate of honeysuckle liquid fermented by LAB on superoxide anion free radical was continuously improved, and the scavenging rate reached the highest value at 24 h of fermentation (Figure 7). Compared with the unfermented, it increased by 27.10, 29.07, and 30.51%, respectively (*p* < 0.05). The honeysuckle liquid using the mixed fermentation agent increased more significantly (*p* < 0.05). During the fermentation process of 24–40 h, the antioxidant capacity of the honeysuckle liquid fermented by LAB gradually became stable, and the free radical clearance rate remained in a certain range.

## 4 Discussion

The honeysuckle beverage was fermented by adding LAB. The sensory quality of honeysuckle beverage was improved, and its health benefits were improved. This is because LAB can decompose macromolecules in food, including the degradation of difficult-to-digest polysaccharides and the conversion of unwanted flavor substances. Therefore, products containing or processed with LAB were considered a natural way to preserve food and promote health (Quinto et al., 2014; Wang et al., 2021). Fermentation temperature and strain addition significantly affected the fermentation of honeysuckle beverage by LAB. LAB ferment sugar in beverages to produce organic acids, especially lactic acid. When the amount of strain increased, the yield of lactic acid increased, which also had a certain effect on the sensory of the fermentation broth. With the increase in the amount of LAB, the taste of the fermentation broth was significantly improved because the lactic acid or other metabolites produced by the fermentation would reduce the bitterness and astringency of the honeysuckle liquid. However, when the concentration of lactic acid is too high, the sensory quality of the product decreases. In the orthogonal experiment, the best process of fermentation of honeysuckle liquid with mixed starter was obtained. The fermentation temperature, fermentation time, the amount of LAB, and sugar were the key factors to determine the quality and nutritional value of LAB-fermented beverages. Different flavor substances are produced in the process of fermentation, which is the difference between LAB-fermented beverage and other fermented beverages.

On this basis, *Lactiplantibacillus plantarum* and *Lactobacillus acidophilus* fermentation were compared with honeysuckle beverage without LAB inoculation, and the physical and chemical indexes and antioxidant activity were analyzed. The number of viable LAB in probiotic products is a key quality indicator, and increasing the number of viable bacteria in the final product is crucial. Honeysuckle contains phenolic acids, flavonoids, triterpenoid saponins, polysaccharides, and volatile oils. These phytochemicals may stimulate the growth of probiotics (Shori et al., 2022), resulting in an increase in the total number of LAB in honeysuckle beverages. To ensure the health benefits of fermented dairy products, probiotics should be at least 10^6^–10^7^ cfu/mL (Shori et al., 2018), which is consistent with the results of this study. The activity of LAB reached more than 10^6^ cfu/mL. The total number of LAB was 9.60 ± 0.08 lg cfu/mL and 9.45 ± 0.04 lg cfu/mL, respectively. A high viable count is essential for acidification, which greatly impacts the sensory quality of fermented beverages (Fessard et al., 2017). During the fermentation process, LAB metabolize to produce organic acids such as lactic acid, so the pH value of honeysuckle liquid showed a downward trend after fermentation. The rapid decrease in pH value is particularly important for the function of probiotics. In foods with a pH value lower than the pH value of the gastrointestinal fluid, it can inhibit the cell growth of harmful bacteria and improve the effect of probiotics by increasing the survival rate of beneficial bacteria (Kailasapathy and Chin, 2000). The increase in total polyphenol content may be attributed to lactic acid fermentation. The total polyphenol content of honeysuckle liquid fermented by LAB was significantly higher than that of honeysuckle liquid without LAB, which was consistent with the experiment (Mantzourani et al., 2018). This is because LAB can convert bound phenols into free phenols. The total flavonoids, polyphenols, and proanthocyanidins in the fruit of *Lonicera japonica* are the main antioxidant components. Many studies have shown that polyphenols have strong antioxidant properties *in vitro* and may help protect cells from oxidative damage caused by free radicals (Gangopadhyay et al., 2016; Tušek et al., 2016). The difference in polyphenol content is one of the important factors affecting the antioxidant activity of honeysuckle beverage. In the study, the content of total polyphenols and total flavonoids in GTK and BTK increased with the extension of fermentation time. The antioxidant capacity of the black tea and green tea fermentation group was higher than that of the unfermented group. The change in antioxidant capacity was consistent with the change in flavonoids and polyphenols. In addition, organic acids and vitamin C were also produced during the fermentation process, resulting in changes in antioxidant capacity (Wang et al., 2022), which was consistent with the results of this study. The synergistic effect between phenolic compounds or with other compounds has a positive impact on the enhancement of antioxidant activity. The results showed that the increase in total polyphenol content after fermentation had a positive effect on DPPH free radical scavenging ability, and the total polyphenol content and DPPH free radical scavenging ability were improved after fermentation (Sabokbar and Khodaiyan, 2016). Ouyang et al. (2018) found that total onion polyphenols showed significant antioxidant activity in DPPH, FRAP, and OH assays. As the total polyphenol concentration increased, the ability to scavenge OH radicals also increased. The results showed that any herb extract could effectively scavenge superoxide *in vitro*, providing useful information for selecting effective antioxidants for food. The polyphenol content clearly reflected its superoxide-scavenging activity (Saito et al., 2008). Beverages fermented with LAB have significantly improved antioxidant activity, and therefore, they have potential health benefits. Finally, it is concluded that the mixed starter culture of *Lactiplantibacillus plantarum* and *Lactobacillus acidophilus* is more suitable for the fermentation of honeysuckle beverage, and its functional quality can be improved by fermentation (Oh et al., 2020). The combination of honeysuckle and probiotics can better meet the beverage requirements under the current big health strategy, and it has certain research significance for enriching China’s fermented beverage market. In addition to the above two LABs, other LAB, yeast, and other beneficial bacteria are beneficial to the fermentation performance and fermentation quality of honeysuckle liquid, and further research is needed to better apply the fermentation technology to the honeysuckle industry.

## 5 Conclusion

In this study, honeysuckle was used as the experimental raw material, and a series of processes were used to make honeysuckle beverage. The optimum conditions of the experiment were obtained by orthogonal experiment: fermentation temperature 37 °C, fermentation time 24 h, *Lactiplantibacillus plantarum* and *Lactobacillus acidophilus* mixed starter (1:1) inoculation amount of 3%, the addition amount of white sugar was 8%. On this basis, the changes in viable count of LAB, pH, total polyphenol content, and antioxidant activity of honeysuckle liquid inoculated with LAB were studied. According to the comprehensive data, the optimized fermented beverage has a soft taste, moderate sour and sweet, and the characteristic flavor of LAB-fermented beverage. The mixed fermentation of *Lactiplantibacillus plantarum* and *Lactobacillus acidophilus* is more suitable for the fermentation of honeysuckle beverage. In future research, the mixed fermentation of honeysuckle extract by more than two kinds of microorganisms can be considered. The synergistic effect of mixed bacterial fermentation was utilized to study the enhancement of nutritional components and flavor substances in honeysuckle beverage. The research results expand the ideas for the deep processing of honeysuckle products and provide process parameters and theoretical basis for the industrial production of honeysuckle-fermented beverage.

## Data availability statement

The original contributions study are included in the article/supplementary material, further inquiries can be directed to the corresponding authors.

## Author contributions

JR: Conceptualization, Data curation, Writing – original draft. YT: Investigation, Writing – original draft. WM: Formal analysis, Methodology, Writing – original draft. XM: Investigation, Methodology, Writing – original draft. LJ: Supervision, Writing – original draft. YL: Formal analysis, Funding acquisition, Writing – review & editing. RZ: Resources, Writing – review & editing. HZ: Data curation, Validation, Writing – original draft.
